# Discrimination of *Tilletia controversa* from the *T. caries*/*T. laevis* complex by MALDI-TOF MS analysis of teliospores

**DOI:** 10.1007/s00253-021-11757-2

**Published:** 2022-01-17

**Authors:** Monika K. Forster, Somayyeh Sedaghatjoo, Wolfgang Maier, Berta Killermann, Ludwig Niessen

**Affiliations:** 1 Institute for Crop Science and Plant Breeding, Bavarian State Research Center for Agriculture (LfL), Voettinger Str. 38, 85354 Freising, Germany; 2 Chair of Microbiology, TUM School of Life Sciences, Technical University of Munich, Gregor-Mendel-Str. 4, 85354 Freising, Germany; 3 Julius Kühn Institute (JKI) – Federal Research Centre for Cultivated Plants, Institute for Epidemiology and Pathogen Diagnostics, Messeweg 11-12, 38104 Braunschweig, Germany; 4 Molecular Phytopathology and Mycotoxin Research, University of Goettingen, Grisebachstrasse 6, 37077 Goettingen, Germany

**Keywords:** Mass spectrometry, Spectral analysis, Common bunt, Dwarf bunt, Morphology, Germination

## Abstract

**Abstract:**

The fungal genus *Tilletia* includes a large number of plant pathogens of *Poaceae*. Only a few of those cause bunt of wheat, but these species can lead to significant yield losses in crop production worldwide. Due to quarantine regulations and specific disease control using appropriate seed treatments for the different disease agents, it is of high importance to distinguish *Tilletia caries* and *Tilletia laevis* as causal agents of common bunt accurately from *Tilletia controversa*, the causal agent of the dwarf bunt. Several studies have shown that matrix-assisted laser desorption/ionization-time of flight mass spectrometry (MALDI-TOF MS) is a useful tool to differentiate closely related fungal species. The aim of this study was to assess whether MALDI-TOF MS analysis is able to distinguish specimens of the three closely related pathogens *T. caries*, *T. laevis*, and *T. controversa* and whether it may constitute an alternative method to the morphology-based identification or germination tests. Spectral data are available via ProteomeXchange with identifier PXD030401. Spectra-based hierarchical cluster analysis (HCA) and discriminant analysis of principal components (DAPC) of the obtained mass spectra showed two main clusters. One cluster included specimens of *T. controversa*, whereas the second cluster comprised *T. laevis* and *T. caries* specimens. Even though main spectral profiles (MSPs) for species identification are missing, MALDI-TOF MS has proven to be a useful method for distinguishing between *T. controversa* and the two causal agents of common bunt, using direct analysis of teliospores, but was unable to separate *T. caries* and *T. laevis* species.

**Key points:**

*• MALDI-TOF MS was developed to classify Tilletia species causing bunt of wheat.*

*• Best results were achieved when combining HCA and DAPC analysis.*

*• The method resulted in an accuracy of 98.51% testing 67 Tilletia specimens.*

**Graphical abstract:**

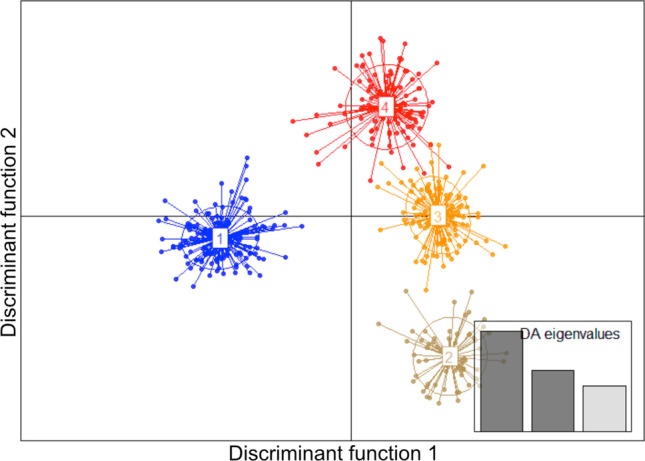

**Supplementary Information:**

The online version contains supplementary material available at 10.1007/s00253-021-11757-2.

## Introduction

More than 170 *Tilletia* species (*Tilletiales*, *Exobasidiomycetes*, *Ustilaginomycotina*) are described and distributed worldwide (Vánky 2012). All species parasitize on inflorescences or leaves of grasses (*Poaceae*). Species are highly diverse in terms of morphology and host specificity. The greater part of them has no economic impact, but a few *Tilletia* species infect economically important cereal crops causing severe yield losses or decreasing the capabilities for further processing due to the production of foul-smelling trimethylamine (Hanna et al. 1932). Wheat is affected by four *Tilletia* species, whereas the causal agents of common bunt of wheat, *Tilletia caries* (DC) Tul. (syn. *T. tritici* (Bjerk.) G. Winter) and *Tilletia laevis* Kühn (syn. *T. foetida* (Wallr.) Liro)), as well as wheat dwarf bunt, *Tilletia controversa* Kühn, are closely related species (Carris et al. 2007). *Tilletia indica* (Mitra), the causal agent of karnal bunt of wheat, is more distantly related (Jayawardena et al. 2019) and has a distinct and limited geographical distribution (Goates 1996). In most areas of the world, the two species *T. indica* and *T. controversa* are categorized as a quarantine pest (Peterson et al. 2009; IPPC 2016; EPPO 2021; https://gd.eppo.int/taxon/TILLCO/categorization). Therefore, efficient and accurate identification methods to discriminate *Tilletia* species on wheat are essential with regard to the global trade of seeds and cereals as well as for targeted treatment of the pathogens.

*Tilletia caries*, *T. laevis*, and *T. controversa* are described as three distinct species, based on their morphological and physiological features (Goates 1996; Vánky 2012). Morphologically, the three *Tilletia* species are determined based on their teliospore characteristics (Hoffmann 1982; Goates 1996; Vánky 2012). However, the morphological distinction of teliospores is challenging because the properties frequently overlap and the morphological variability of the teliospores can be quite high (Holton and Kendrick 1956; Holton et al. 1968). It is important to use mature teliospores for the identification of the *Tilletia* species as morphology differs with teliospore age (Durán and Fischer 1961). Because of extensive variation in the morphological as well as in several physiological and genetic traits, it was also suggested that the three species might be conspecific (Russell and Mills 1994). Phylogenetic and phylogenomic studies could not resolve the three species, respectively (Carris et al. 2007; Sedaghatjoo et al. 2021b, submitted). The germination behavior of teliospores of *T. caries*, *T. laevis*, and *T. controversa* correlates with the disease symptoms they are causing. The causal agents of common bunt, *T. caries* and *T. laevis*, start to germinate at 14–16 °C within 4 to 5 days, but also germinate at 5 °C, needing roughly double the time (Lowther 1950). By contrast, teliospores of *T. controversa* only germinate at 5 °C within 4 to 6 weeks (Meiners and Waldher 1959) and no germination occurs at higher temperatures. However, germination testing is unable to distinguish between *T. caries* and *T. laevis*, observation of the germination behavior of teliospores was found to be the most reliable approach to differentiate teliospores of common and dwarf bunt of wheat (Trione and Krygier 1977). Nevertheless, this classification method is very time-consuming and not applicable in official seed testing.

Several studies have attempted the detection of the wheat bunt species using PCR or other DNA-based methods. But, most of these assays are not able to distinguish between common (*T. caries*/*T. leavis*) and dwarf bunt (*T. controversa*). In particular, none of the assays is able to differentiate the two common bunt taxa *T. caries* and *T. laevis* (Mulholland and McEwan 2000; Josefsen and Christiansen 2002; Kochanová et al. 2004; Zouhar et al. 2010; Pieczul et al. 2018). Other assays designed to specifically detect *T. controversa* were only tested against a relatively small number of specimens but it was not shown if the specificity applies to a high number of specimens with different origins (Liu et al. 2009, 2020; Gao et al. 2010, 2011, 2014).

An approach that analyzed protein patterns of *T. caries* and *T. controversa* extracts with electrophoresis methods was applied to determine species-specific differences (Banowetz et al. 1984; Weber and Schauz 1985). Kawchuk et al. (1988) found 359 polypeptides that were common among *T. caries*, *T. controversa*, and *T. laevis*, but the authors were unable to correlate the remaining 13% of uncommon polypeptides with morphological or physiological properties of the three species. In another analysis, an abundant 116-kD polypeptide of undefined nature was detected in extracts from teliospores of *T. controversa* exclusively (Banowetz and Doss 1994). Numerous research groups tried to develop immunoassays to differentiate the three *Tilletia* species, but were unable to create assays specific to all three of them (Eibel et al. 2005; Zhang et al. 2007; Gao et al. 2015). Innovative proteomic analyses of *Tilletia* species were performed by Li et al. (2018) using the isobaric tags for relative and absolute quantitation (iTRAQ) technique to explore proteomic differences among *T. controversa*, *T. caries*, and *T. laevis* by detecting relative quantities of specific proteins between the pathogens and Pandey et al. (2018 and 2019) applied tandem mass spectrometry to identify pathogenicity or virulence-related proteins of *T. indica*.

In several studies, matrix-assisted laser desorption/ionization time-of-flight mass spectrometry (MALDI-TOF MS) analysis of fungal subproteomes was introduced as a useful and rapid tool to identify and classify microorganisms (Chalupová et al. 2014). This approach was originally used for bacterial identification (Welham et al. 1998). Li et al. (2000) and Welham et al. (2000) were the first to transfer the application from bacteria to spores of clinically relevant filamentous fungi. Increasing numbers of protocols have been published to analyze fungal spores by MALDI-TOF MS for a broad variety of fungi and oomycetes, including many phytopathogens (Böhmer et al. 2007; Kemptner et al. 2009; Sulc et al. 2009; Bhadauria et al. 2010; Brun et al. 2013; Beinhauer et al. 2016). The technique was also applied to distinguish closely related species (Gruenwald et al. 2015; Wigmann et al. 2019) and could therefore also be a useful tool for the discrimination of the wheat bunt fungi *T. caries*, *T. laevis*, and *T. controversa*. The purpose of the current study was to develop and test a protocol for the MALDI-TOF MS-based analysis of *Tilletia* spp. teliospores for a fast and reliable distinction of the bunt pathogens of wheat.

## Material and methods

### *Tilletia* teliospore material

The 69 analyzed specimens of *T. caries*, *T. laevis*, *T. controversa*, and *T. indica*, their geographic origins, and voucher information are listed in Table 1. The infected spikelets and individual bunt balls of *T. caries*, *T. laevis*, and *T. controversa* originated mainly from Europe and the USA, while two specimens of *T. controversa* were originally collected in Turkey, but maintained through wheat infections in the USA (Goates 2012). The two *T. indica* specimens originated from India and Mexico. All specimens were stored as intact bunt balls, mainly in the ears of wheat, at 10 °C and weakly humid conditions at the official seed testing laboratory of the Bavarian State Research Center for Agriculture (LfL, Freising, Germany).

**Table 1:** List of specimens used in this study and their classification with different methods

Number	Sample number^1^	Collection year^2^	Host	Origin^3^	Collector/source	Classification germination	HCA cluster	DAPC cluster	Classification MALDI-TOF MS	Classification morphology	Final classification
1	AA7	2015	*T. aestivum*^*4*^	AUT	A. E. Müllner	Cb^5^	2	3	Cb	*T. caries*	Cb
2	AA8	2015	*T. aestivum*	AUT	A. E. Müllner	Cb	2	3	Cb	*T. caries*	Cb
3	AA9	2015	*T. aestivum*	AUT	A. E. Müllner	Cb	2	3	Cb	*T. caries*	Cb
4	AA10	2015	*T. aestivum*	AUT	A. E. Müllner	Cb	2	3	Cb	*T. caries*	Cb
5	AA11 (CBS 144,825)^6^	2015	*T. aestivum*	AUT	A. E. Müllner	Cb	2	3	Cb	*T. caries*	Cb
6	AA12	2015	*T. aestivum*	AUT	A. E. Müllner	Cb	2	3	Cb	*T. caries*	Cb
7	AC	2015	*T. aestivum*	DEU	H. Spieß	Cb	2	3	Cb	*T. caries*	Cb
8	AD	2014	*T. aestivum*	DEU	S. Schumann	Cb	2	3	Cb	*T. caries*	Cb
9	AER	2016	*T. aestivum*	DEU	S. Weller	Cb	2	3	Cb	*T. caries*	Cb
10	AES	2016	*T. aestivum*	DEU	S. Weller	Cb	2	3	Cb	*T. caries*	Cb
11	AEZO	2016	*T. spelta*	DEU	S. Weller	Cb	2	3	Cb	*T. caries*	Cb
12	AGW	2016	*T. aestivum*	DEU	B. Schwab	Cb	2	3	Cb	*T. caries*	Cb
13	AHW	2016	*T. aestivum*	DEU	S. Weller, H. Eichinger	Cb	2	3	Cb	*T. caries*	Cb
14	AI (CBS 145,171)	2015	*T. durum*	ITA	V. Weyermann	Cb	2	3	Cb	*T. laevis* > *T. caries*^7^	Cb
15	AKW	2016	*T. aestivum*	DEU	S. Weller	Cb	2	3	Cb	*T. caries*	Cb
16	AL	2010	*T. aestivum*	DEU	H. Mitterer	Cb	2	4	Cb	*T. caries*	Cb
17	AL14	2014	*T. aestivum*	DEU	H. Mitterer	Cb	2	4	Cb	*T. caries* > *T. controversa*	Cb
18	AL15	2015	*T. aestivum*	DEU	H. Mitterer	Cb	2	4	Cb	*T. caries*	Cb
19	AN	2014	*T. aestivum*	DEU	R. Bauer	Cb	2	4	Cb	*T. caries*	Cb
20	AN15	2015	*T. aestivum*	DEU	R. Bauer	Cb	2	4	Cb	*T. caries*	Cb
21	AO (CBS 145,172)	2014	*T. aestivum*	DEU	R. Bauer	Cb	2	4	Cb	*T. caries*	Cb
22	AOA	2016	*T. aestivum*	DEU	B. Schwab	Cb	2	3	Cb	*T. caries* > *T. controversa*	Cb
23	ARW	2016	*T. aestivum*	DEU	S. Weller	Cb	2	3	Cb	*T. caries* > *T. laevis*	Cb
24	AS14	2014	*T. aestivum*	DEU	B. Pölitz	Cb	2	3	Cb	*T. caries*	Cb
25	AW	2014	*T. aestivum*	DEU	R. Bauer	Cb	2	3	Cb	*T. caries*	Cb
26	AZH1	2015	*T. aestivum*	CHE	V. Weyermann	Cb	1 (b)^8^	3	Cb	*T. caries* > *T. laevis*	Cb
27	AZH2	2015	*T. aestivum*	CHE	V. Weyermann	Cb	1 (b)	2	Cb	*T. caries* > *T. laevis*	Cb
28	AZH3 (CBS 145,166)	2015	*T. aestivum*	CHE	V. Weyermann	Cb	1 (b)	1 (b)	Db	*T. caries* > *T. laevis*	Cb
29	AZH4	2015	*T. aestivum*	CHE	V. Weyermann	Cb	2	3	Cb	*T. caries* > *T. controversa*	Cb
30	AZH5	2015	*T. aestivum*	CHE	V. Weyermann	Cb	2	3	Cb	*T. caries* > *T. controversa*	Cb
31	D-3	-^9^	*T. aestivum*	USA	R. J. Metzger, J. A. Hoffmann	Db^10^	1	4 (b)	Db	*T. controversa*	Db
32	D-4	-	*T. aestivum*	USA	R. J. Metzger, J. A. Hoffmann	Db	1	4 (b)	Db	*T. controversa*	Db
33	D-7	1999 (a)	*T. aestivum*	USA	R. J. Metzger, J. A. Hoffmann	Db	1	1	Db	*T. caries* > *T. controversa*	Db
34	D-12	-	*T. aestivum*	USA	R. J. Metzger, J. A. Hoffmann	Db	1	1	Db	*T. controversa*	Db
35	D-13	-	*T. aestivum*	USA	R. J. Metzger, J. A. Hoffmann	Db	1	1	Db	*T. controversa*	Db
36	D-17	1999 (a)	*T. aestivum*	USA	R. J. Metzger, J. A. Hoffmann	Db	1	1	Db	*T. controversa*	Db
37	D-18	-	*T. aestivum*	USA	B. J. Goates, R. J. Metzger	Db	1	1	Db	*T. controversa*	Db
38	D-19	-	*T. aestivum*	TUR	B. J. Goates, R. J. Metzger	Db	1	1	Db	*T. controversa* > *T. caries*	Db
39	II7	2007	*T. spp.*	IND	P. Chhuneja	NA^11^	outgroup	outgroup	Kb^12^	*T. indica*	Kb
40	IM5	2005	*T. spp.*	MEX	G. Fuentes	NA	outgroup	outgroup	Kb	*T. indica*	Kb
41	L-1	1990 (a)	*T. aestivum*	-	R. J. Metzger, J. A. Hoffmann	ng^13^	0 (b)	2	Cb	*T. laevis*	Cb
42	L-10	1990 (a)	*T. aestivum*	-	R. J. Metzger, J. A. Hoffmann	Cb	2	2	Cb	*T. laevis*	Cb
43	L-16	1984 (a)	*T. aestivum*	-	R. J. Metzger, J. A. Hoffmann	Cb	2	2	Cb	*T. laevis*	Cb
44	L-18	-	*T. aestivum*	-	R. J. Metzger	Cb	2	2	Cb	*T. laevis*	Cb
45	L-19 (CBS 145,173)	-	*T. aestivum*	-	R. J. Metzger	Cb	2	2	Cb	*T. laevis*	Cb
46	L-20	-	*T. aestivum*	TUR	R. J. Metzger	Cb	2	2	Cb	*T. laevis*	Cb
47	L-21	-	*T. aestivum*	USA	R. J. Metzger	Cb	2	2	Cb	*T. laevis*	Cb
48	OA1	2015	*T. aestivum*	AUT	A. E. Müllner	Db	1	1	Db	*T. controversa*	Db
49	OA2 (CBS 145,169)	2015	*T. aestivum*	AUT	A. E. Müllner	Db	1	1	Db	*T. controversa*	Db
50	OA3	2015	*T. aestivum*	AUT	A. E. Müllner	Db	1	1	Db	*T. controversa*	Db
51	OA4	2015	*T. aestivum*	AUT	A. E. Müllner	Db	1	1	Db	*T. controversa* > *T. caries*	Db
52	OA5	2015	*T. aestivum*	AUT	A. E. Müllner	Db	1	1	Db	*T. controversa*	Db
53	OA6	2015	*T. aestivum*	AUT	A. E. Müllner	Db	1	1 / 4 (bc)^14^	Db	*T. controversa*	Db
54	OC1	2015	*T. aestivum*	DEU	H. Spieß	Db	1	1	Db	*T. controversa*	Db
55	OC2	2015	*T. aestivum*	DEU	H. Spieß	Db	1	1	Db	*T. controversa*	Db
56	OL	2013	*T. aestivum*	DEU	H. Mitterer	Db	1	1	Db	*T. controversa*	Db
57	OL14 (CBS 145,167)	2014	*T. aestivum*	DEU	H. Mitterer	Db	1	1	Db	*T. controversa*	Db
58	OL16	2016	*T. aestivum*	DEU	M. K. Forster	Db	1	1	Db	*T. controversa*	Db
59	OMO	2016	*T. spelta*	DEU	R. Klügl	Db	1	1	Db	*T. controversa*	Db
60	OR (CBS 144,827)	2013	*T. aestivum*	DEU	R. Bauer	Db	1	1	Db	*T. controversa*	Db
61	ORB	2016	*T. aestivum*	DEU	S. Weller	Db	1	1	Db	*T. controversa*	Db
62	OV (CBS 145,170)	2011	*T. aestivum*	DEU	R. Bauer	Db	1	1	Db	*T. controversa*	Db
63	OW15	2015	*T. aestivum*	DEU	R. Bauer	Db	1	1	Db	*T. controversa*	Db
64	T-2	1989 (a)	*T. aestivum*	USA	R. J. Metzger, J. A. Hoffmann	Cb	2	2	Cb	*T. caries*	Cb
65	T-15	1978 (a)	*T. aestivum*	USA	R. J. Metzger, J. A. Hoffmann	Cb	2	4	Cb	*T. caries*	Cb
66	T-19	-	*T. aestivum*	USA	R. J. Metzger, J. A. Hoffmann	Cb	2	2	Cb	*T. caries*	Cb
67	T-30	-	*T. aestivum*	USA	R. J. Metzger, J. A. Hoffmann	Cb	2	2	Cb	*T. caries* > *T. laevis*	Cb
68	T-33	-	*T. aestivum*	USA	R. J. Metzger	Cb	2	2	Cb	*T. caries*	Cb
69	T-34	-	*T. aestivum*	USA	R. J. Metzger	Cb	2	2	Cb	*T. caries* > *T. laevis*	Cb

^1^Sample numbers/collection numbers are sorted alphabetically, sample number of *T. caries* specimens start with A or T, *T. controversa* specimens starts with D or O, *T. indica* specimens with I, and *T. laevis* specimens starts with L

^2^(a) Year of original collection, samples constantly recultivated until 2012 by B. J. Goates

^3^International abbreviations used: *AUT*, Austria; *CHE*, Switzerland; *DEU*, Germany; *IND*, India; *ITA*, Italy; *MEX*, Mexico; *TUR*, Turkey; *USA*, United States of America

^4^*T*. = *Triticum*

^5^*Cb*, common bunt

^6^CBS = single teliospore culture produced and stored at strain collection of Westerdijk Fungal Biodiversity Institute, Utrecht, The Netherlands

^*7*^*T. laevis* > *T. caries* = *T. laevis* rather than *T. caries*

^8^(b) = false classification

^9^No information available

^10^*Db*, dwarf bunt

^11^*NA*, not analyzed

^12^*Kb*, karnal bunt

^13^*ng*, no germination

^14^(bc) = false classification (*N* = 67 + outgroup), true classification (*N* = 52 + outgroup, reference samples)

### Morphological determination of the bunt species

For morphological identification of the species, teliospores of each specimen were isolated from intact bunt balls, embedded in Hoyer’s fluid (Cunningham 1972), and determined using light microscopy considering the main differentiation criteria shape and degree of reticulation of the teliospores (ISTA 1984; Vánky 2012). The examination was performed independently by seven scientists, all experienced in the morphological determination of bunts of wheat.

### Classification by germination behavior and culture conditions

To determine species group identification by germination behavior, two units of approximately hundreds of thousands to one million teliospores of all *T. caries*, *T. laevis*, and *T. controversa* specimens were surface sterilized using 0.26% aqueous solution of sodium hypochlorite and rinsed twice in sterile water (Wilcoxson and Saari 1996). Subsequently, the teliospores were resuspended in 500 μl sterile water and streak inoculated on 2% water agar. Each specimen was incubated at 5 °C and 15 °C, respectively, for up to 6 weeks. Samples were controlled daily under the light microscope to detect the starting time of germination and to calculate the germination rate. Following the definition of Schauz (1968), a *Tilletia* teliospore was judged as germinated when the hypha was at least as long as the diameter of the teliospore. This was repeated twice for each specimen.

Two specimens, *T. caries* (AL15) and *T. controversa* (OL16), were selected in this study to implement and optimize the method. A single germinated teliospore of these *Tilletia* specimens was transferred to M-19 agar media (Trione 1964) and cultivated, maintained, and lyophilized as described by Sedaghatjoo et al. (2021a) to obtain enough biomass for MALDI-TOF MS analyses. Lyophilized mycelium of specimens AL15 and OL16 were stored at the Federal Research Centre for Cultivated Plants (JKI, Braunschweig, Germany).

### Sample preparation for MALDI-TOF MS analysis

Samples were prepared from teliospores and lyophilized mycelium. For teliospore isolation, bunt balls were opened carefully on weighing paper (Macherey–Nagel GmbH, Düren, Germany) using a pair of fine forceps. The wheat tissue was removed and the teliospores were transferred to a 2-ml reaction tube (Eppendorf, Hamburg, Germany). Three milligrams of lyophilized mycelia or teliospores, respectively, was transferred into a 1.5-ml reaction tube (Eppendorf, Hamburg, Germany). Surface sterilization and inactivation of the fungal cells was performed by resuspending in 300 μl sterile deionized water and subsequently applying 900 μl of ethanol (VWR International, Fontenay-sous-Bois, France). Samples were vortexed and centrifuged (Andreas Hettich GmbH, Tuttlingen, Germany) for 10 min at 4 °C, 21,380 × *g*. The supernatant was carefully removed by pipetting and the pellet was dried completely in a laminar flow cabinet for 30 min (teliospores) or in a vacuum desiccator at 5 mbar (mycelium) with the lids of the reaction tubes open. Extraction of the proteins from the mycelium was performed according to the ethanol/formic acid extraction sample preparation protocol for microorganism (Bruker Daltonik GmbH 2011). The procedure was slightly modified to extract the proteins from teliospores. These were first suspended in 100 μl of formic acid (70%) (Sigma-Aldrich Chemie, Steinheim, Germany) and then transferred to innuSPEED Lysis Tube X (2 ml) (Analytik Jena, Jena, Germany) containing sterile ceramic beads of different sizes (0.4–0.6 mm & 1.4–1.6 mm). Homogenization of the samples was conducted in a FastPrep®-24 (MP Biomedicals, Eschwege, Germany) at 6.0 m/s for 40 s. After the addition of 100 μl of acetonitrile (100%) (Sigma-Aldrich Chemie, Steinheim, Germany), samples were vortexed for 5 min at maximum speed. The suspension was transferred into a new 1.5-ml reaction tube without ceramic beads. Samples were centrifuged for 2 min at room temperature at 20,000 × *g*. A MALDI 96 polished steel target plate (Bruker Daltonics, Bremen, Germany) was prepared by applying 1 μl of matrix solution containing 10 mg/ml α-cyano-4-hydroxy-cinnamic acid in acetonitrile, deionized water, and trifluoroacetic acid (50:47.5:2.5, v/v/v) (Sigma-Aldrich Chemie, Steinheim, Germany). One microliter of the cell-free supernatant was transferred on top of the air-dried matrix spot, allowed to air dry at room temperature, and subsequently overlaid by another 1 μl of matrix solution before being air-dried again at ambient temperature. Three replicates of each sample were prepared and analyzed.

### MALDI-TOF mass spectra collection and data processing

Mass spectra were generated using a Microflex LT MALDI-TOF mass spectrometer (Bruker Daltonics, Bremen, Germany) equipped with a nitrogen laser (*λ* = 337 nm) recording spectra in linear positive ion detection mode at a laser frequency of 20 Hz within a mass range from 2000 to 20,000 Da. The software used for data acquisition was MALDI Biotyper 3.0 Realtime classification (RTC) (Bruker Daltonics, Bremen, Germany) and FlexControl 3.4 (Bruker Daltonics, Bremen, Germany). Parameter settings were 20.11 kV (ion source 1), 18.86 kV (ion source 2), 6.53 kV (lens), and pulsed ion extraction 230 ns. The laser power was adjusted to 40% with an attenuator range of 30% and an offset of 23%. Each MALDI-TOF mass spectrum was recorded by 40-shot steps from random positions of the target spot, summarized to 240 single spectra.

The software FlexAnalysis 3.4 (Bruker Daltonics, Bremen, Germany) was used for visual inspection of the recorded mass spectra. Raw data was converted to a text file, listing intensities versus *m/z* data points spaced 0.25 Da from each other. Preprocessing steps like subtracting the baseline, smoothing and normalizing signal intensities of the single mass spectra, peak picking, and the calculation of the signal to noise ratio (SNR) based on the noise level for each m/z value were performed by using mass spectrometry comparative analysis package (MASCAP) (Mantini et al. 2010), implemented in the GNU Octave (3.8.1) software package (https://www.gnu.org/software/octave/), according to Schott et al. (2016). For further analysis and comparison, peaks of all extracted spectra were calibrated and aligned considering a tolerant peak shift range of 600 ppm of the *m/z* value to define peaks to be identical (Wang et al. 2006; Usbeck et al. 2014).

The reproducibility of the spectra extraction method was verified using MASCAP. Three biological replicates of each sample were spotted in triplicate onto the MALDI stainless polished steel target to obtain nine single spectra per specimen. These were summarized as one mean spectrum and illustrated in a stacked view. This visualization of MALDI-TOF MS spectra profiles was also used to compare the peak profiles of teliospores and mycelium of the reference specimens AL15 and OL16. The mass spectrometry proteomics data were deposited on the ProteomeXchange Consortium platform (http://proteomecentral.proteomexchange.org/cgi/GetDataset) (Deutsch et al. 2020) via the PRIDE (Perez-Riverol et al. 2019) partner repository and can be retrieved with the dataset identifier PXD030401.

### MALDI-TOF MS data analysis

To analyze the subproteome fingerprints of the *Tilletia* species, two different approaches were conducted. The first one was used to compare the mass spectra to each other by high-throughput multidimensional scaling (HiT-MDS, https://sourceforge.net/projects/hitmds/) (Strickert et al. 2005) implemented in the GNU Octave (3.8.1) software package, together with hierarchical cluster analysis (HCA). The second approach was performed by discriminant analysis of principal components (DAPC) using the adegenet package (2.0.1) for the RStudio software (1.1.463) (Jombart and Collins 2015). Both procedures were first conducted using a reference set of 52 *Tilletia* specimens that had been determined to species level without any conflicts by experts. They were then repeated using these and the additional 15 specimens that had been proven to be problematic to determine to species level based on morphological characters (see Table 2).

**Table 2:**
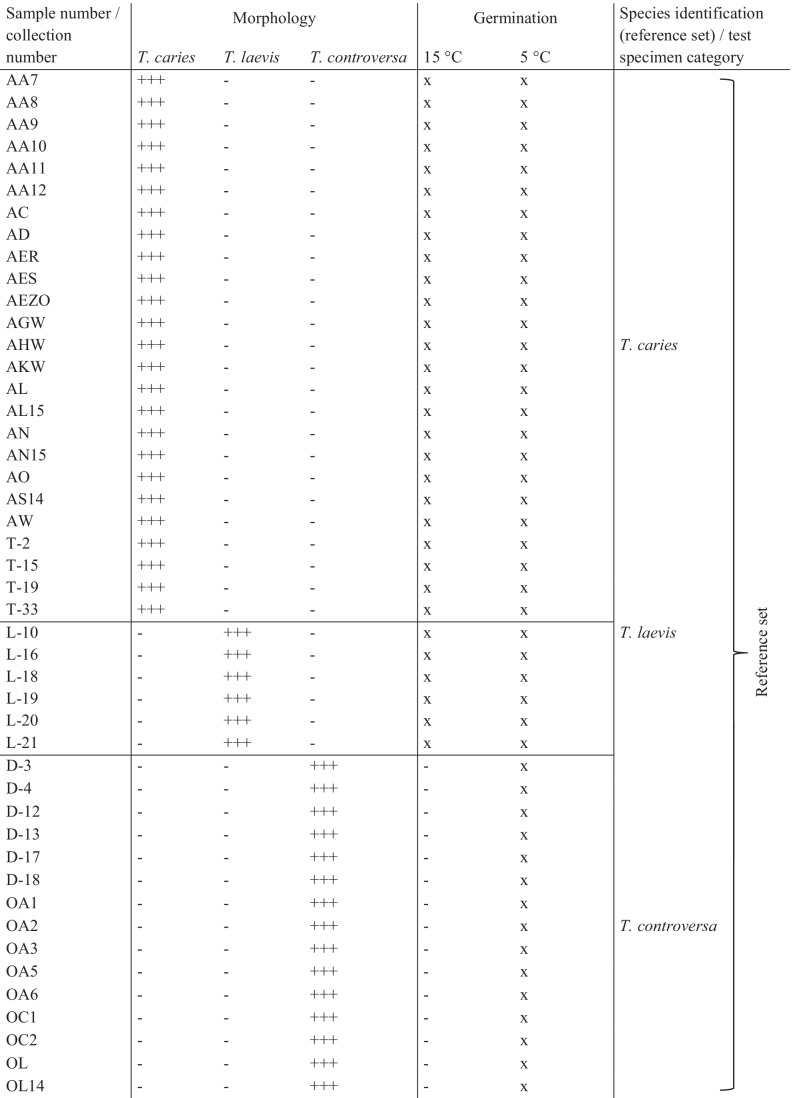

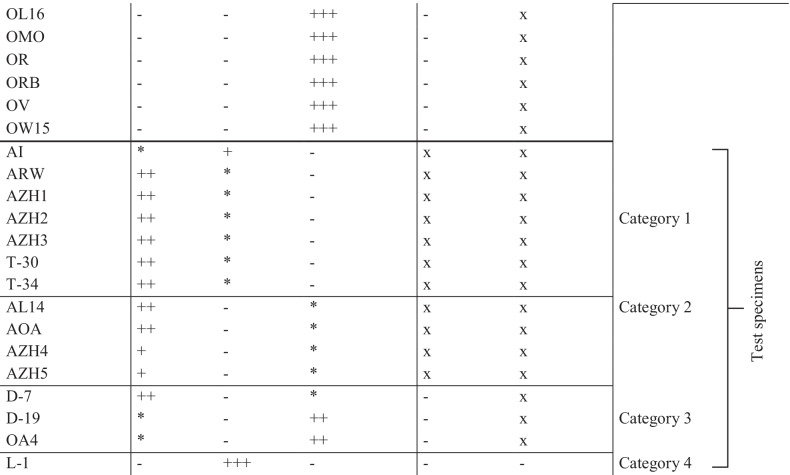
Morphological discrimination and germination behavior of 67 specimens of *T. caries*, *T. laevis*, and *T. controversa*

+  +  + , identically identified by seven experts; +  + , predominantly identified by 5 or 6 experts; + , predominantly identified by 4 experts; *, alternative species identification by minority group; x, triplicates germinated at corresponding temperature; − , negative

HiT-MDS was used to detect similarities and calculate the distances between the acquired mass spectra of the *Tilletia* specimens and to visualize this in a dendrogram. To decrease the complexity of the diagram, consensus spectra were used, where the nine single spectra of each sample were summarized to one mean spectrum. Using an in-house software based on MASCAP, cluster analysis of the condensed mass spectra was performed considering the weighted pair group method with averaging (WPGMA) (Sneath and Sokal 1973) and the normalized dot product to find similarities by comparing the spectra to each other, as described by Frank et al. (2008). The separation of the groups in the resulting dendrogram can be indicated by the reconstruction quality ranging from 0 to 1, where a good separation is represented by 1 and no separation by 0.

The second approach, DAPC, was used to detect and visualize the differences of the mass spectra between groups, instead of the similarities, minimizing the variance within the clusters, which is the main difference to the more commonly used principal components analysis (PCA) method (Jombart and Collins 2015). For best results considering the variability of the mass spectra, all individual single spectra of the specimens were applied. Using the adegenet package (2.0.1) for the RStudio software (1.1.463) (Jombart and Collins 2015), the raw data were first transformed using PCA, followed by applying a k-means algorithm with increasing values of k to identify the optimal number of clusters. Bayesian information criterion (BIC) was used to compare the different clustering solutions where the best one corresponds to the lowest BIC. In this study, the first conspicuous bend in the curve was considered optimal BIC. After choosing the number, DAPC was performed resulting in a bar plot of eigenvalues, a scatterplot which visualizes the individual samples as dots and groups as inertia ellipses, and a table listing the single spectra of each specimen per group. Grouping rules of DAPC demand that a minimum of six out of nine single spectra (66.67%) must cluster together in the same group to reliably assign a specimen to a group. In this study, also specimens only clustering the majority of the nine single spectra to one single group were considered accordingly and marked in Table 3. The main peaks responsible for the separation (of the specimens) were illustrated in a loading plot. To discover the main differences between the two groups in more detail, the DAPC was also performed pairwise based on the grouping by the main DAPC.

**Table 3:** List of DAPC-based grouping of 52 *Tilletia* reference specimens and all 67 *Tilletia* specimens including 15 test specimens regarding the scatterplots in Fig. 5a and b

Group 1		Group 2		Group 3		Group 4	
Reference specimens	+ Test specimens	Reference specimens	+ Test specimens	Reference specimens	+ Test specimens	Reference specimens	+ Test specimens
D-12^a^	D-12	L-10	L-10	AA7	AA7	AL	AL
D-13	D-13	*L-16 (4,3)*^d^	L-16	AA8	AA8	AL15	AL15
D-17	D-17	L-18	L-18 (4)	AA9	AA9	AN	AN
D-18	D-18	L-19	L-19	AA10	AA10	AN15	AN15
OA1	OA1 (4)	L-20	L-20	AA11	AA11	AO (3)	*AO (2,3)*
OA2	OA2 (4,2)	L-21	L-21	AA12	AA12	D-3	D-3
OA3	OA3	T-2	T-2	AC	AC	D-4	D-4
OA5	OA5	T-19 (4,3)	T-19 (3,4)	AD	AD	T-15	T-15 (2)
OA6 (4)^b^	-	T-33 (4)	T-33	AER	AER		**OA6 (1,2)**
OC1	OC1		**AZH2**	AES	AES		**AL14**
OC2	OC2		**L-1**	AEZO	AEZO		
OL	OL		**T-30**	AGW (4)	AGW		
OL14	OL14		**T-34**	AHW	AHW		
OL16	OL16			AKW	AKW		
OMO	OMO			AS14 (4)	AS14		
OR	OR			AW	AW		
ORB	ORB				**AI (4)**		
OV	OV				**AOA**		
OW15	OW15				**ARW**		
	**AZH3 (3)**^**c**^				**AZH1 (1,4)**		
	**D-7**				**AZH4**		
	**D-19**				**AZH5 (4,2)**		
	**OA4**						

^a^Specimens complying with DAPC grouping rules by clustering a minimum six out of nine single spectra (66.67%) together in one group are unmarked

^b^Specimens clustering five single spectra (55.55%) to one group (majority group). Allocation of up to four single spectra to other groups (minority group) are given in brackets by decreasing order

^c^15 test specimens are bold

^d^Two specimens presented in italics had only four spectra in the majority group and five spectra in two other groups

### Classification and accuracy of species discrimination by the developed MALDI-TOF MS method

To determine the final species classification by MALDI-TOF MS, the results of both HCA and DAPC approaches were combined. These composite classification results were compared to the species determination results obtained by morphology and germination behavior. The accuracy of the MALDI-TOF MS method was then calculated as follows:

$$Accuracy\ [\mathrm{\%}]= (correct\ species\ classification/total\ tested\ specimens) \times 100$$

## Results

### Defining reference material and test specimens by morphology and germination behavior

From the 67 specimens of *T. caries*, *T. laevis*, and *T. controversa* studied, 25 were unambiguously determined based on their morphological characteristics as *T. caries*, 7 as *T. laevis*, and 21 as *T. controversa* (see Table 2). Germination behavior was used as a second character for the identification of specimens. Teliospores of 43 specimens germinated at 5 °C as well as at 15 °C and were, based on this criterion, defined as one of the two causal agents of common bunt, *T. caries* or *T. laevis*. The remaining 24 specimens germinated exclusively at 5 °C and were thus defined as *T. controversa*. Combining the results, we defined a reference set of 52 specimens which could clearly be attributed to one of the three *Tilletia* species and this collection was subsequently used to optimize the MALDI-TOF MS. Each of these specimens showed both the species-specific germination behavior as well as the typical morphological characteristics. Within the common bunt species, *T. caries* and *T. laevis* were indistinguishable in their germination behavior but had a distinct morphology. Fourteen further specimens could not be determined unequivocally by their morphological characteristics and one did not germinate. These inconsistently determined specimens (*n* = 15) were defined as the set of test specimens to be identified during the current study and categorized in four different groups as follows (see Table 2): seven specimens were grouped in category 1, which contains six specimens that morphologically were predominantly identified as *T. caries* by five to six experts with a low identification score for *T. laevis*, while one specimen was identified as *T. laevis* by four and as *T. caries* by three experts. The germination behavior supported that the specimens belonged to one of the two causal agents of common bunt – *T. caries* or *T. laevis*. Category 2 contained four specimens that were morphologically identified as *T. caries* by the majority of the experts but had also a low identification score for *T. controversa*, albeit germination tests identified them as one of the common bunt species *T. caries* or *T. laevis.* Category 3 contained three specimens with low identification scores both in *T. caries* and *T. controversa* with germination results that clearly identified them as *T. controversa*. One specimen that was clearly identified morphologically as *T. laevis*, but did not germinate at any temperature regime, was assigned to category 4.

### Differentiation of *T. caries* and *T. controversa* by mass spectra comparison

**Fig. 1:**
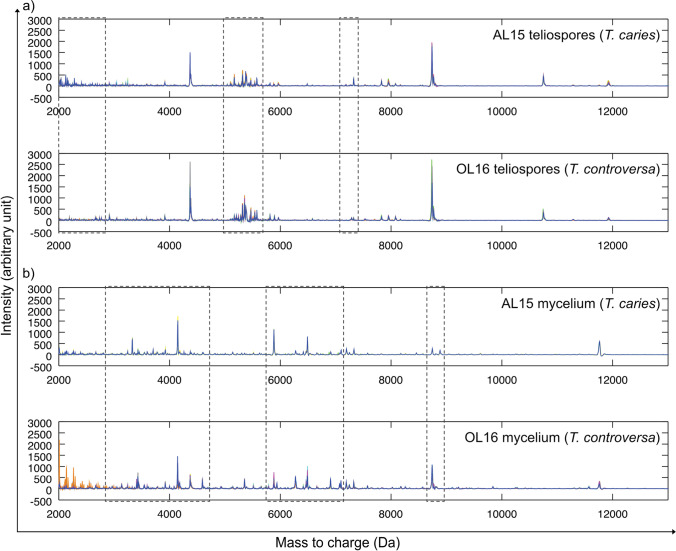
Stacked view of MALDI-TOF MS spectra visualizing the comparison of profiles of *T. caries* specimen AL15 and *T. controversa* specimen OL16 obtained by MALDI-TOF mass spectrometry using **a** teliospores as the starting material, **b** mycelium as the starting material. Overlapping peaks that were identical in location and intensity in all nine single spectra of a stack are shown in blue color, whereas peaks that occurred only in individual spectra or had divergent intensities are visualized in other colors. The parts of the mass spectra that were different between the two specimens are highlighted with boxes

Figure 1a  shows a stack of nine single spectra obtained from teliospores of both strains, whereas Fig. 1b shows a stack of nine single spectra obtained from mycelia of both specimens using a similar preparation method. Comparing the teliospore-based spectra of both specimens showed major differences between individual spectra within three *m/z* ranges, i.e., in the *m/z* range of 2000 to 2700, 5000 to 5500, and around 7300. The major peak differences between single spectra of mycelial preparations of specimens AL15 and OL16 were located in three *m/z* ranges between 3000 and 4500 as well as 5900 and 7100 and around 8900.

**Fig. 2:**
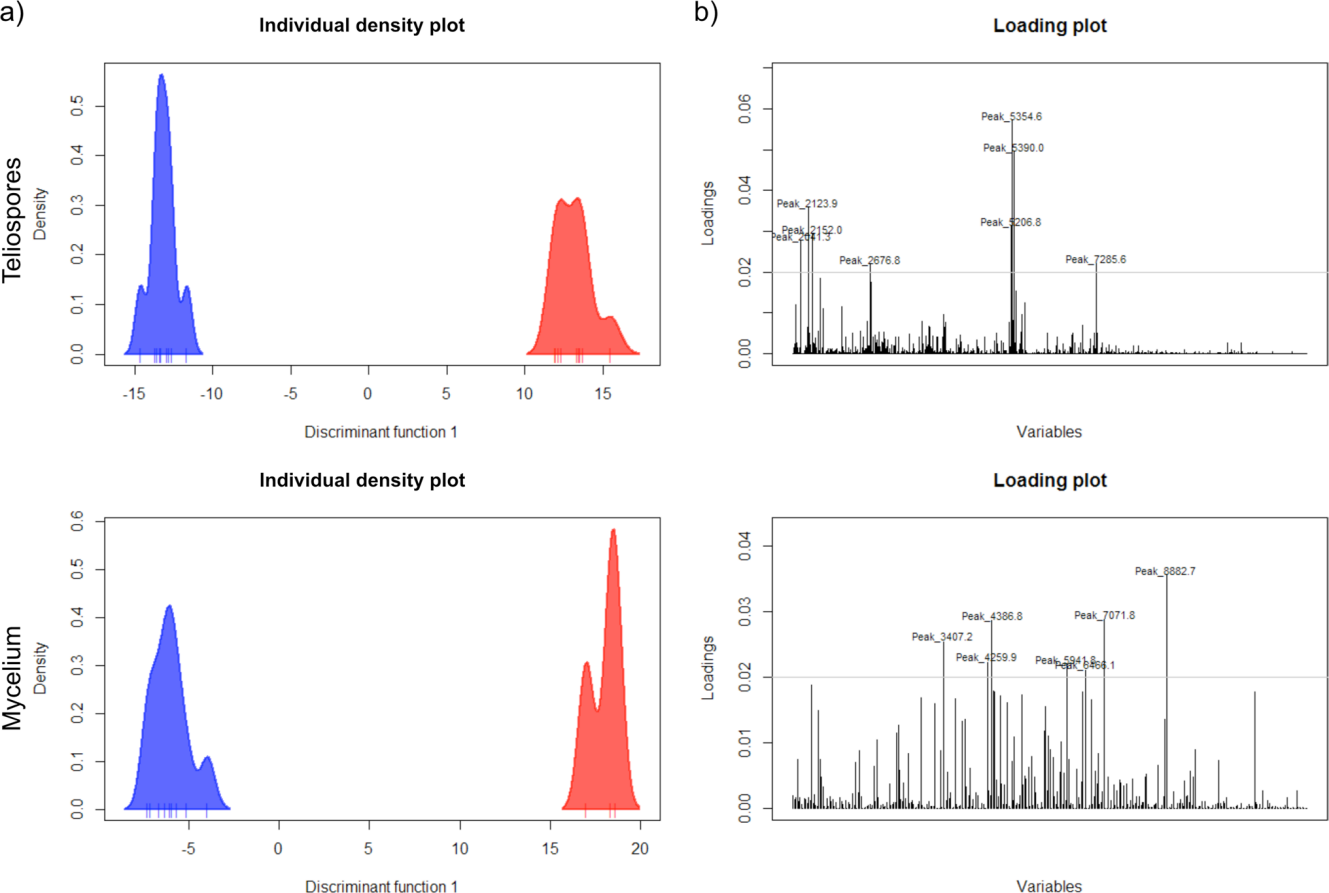
Illustration of discriminant analysis of principal components (DAPC) performed with each nine single MALDI-TOF mass spectra. **a** Individual density plot of teliospores (top) and mycelium (bottom) of the two specimens AL15 (red, right) and OL16 (blue, left); **b** loading plot of single spectra comparison showing peaks used for differentiation between AL15 and OL16 (top teliospores, bottom mycelium)

The individual density plots shown in Fig. 2a resulted from the DAPC analysis of all the single mass spectra (*n* = 9) obtained for each specimen. It showed a clear separation between *T. controversa* (blue, left side) and *T. caries* (red, right side). This difference occurred both in teliospore-based spectra and in mycelium-based spectra. Additionally, the narrowness of the density plot per specimen indicated a high degree of similarity of the nine single spectra within each specimen, both with teliospores and mycelia. All differentiation peaks between *T. caries* and *T. controversa* obtained by DAPC exceeding the threshold of 0.02 were visualized by loading plots and labeled with their *m/z* value (see Fig. 2b). Data corresponded well with the *m/z* areas that were highlighted in Fig. 1.

### Clustering of reference material and test specimens using hierarchical cluster analysis of MALDI-TOF MS data

**Fig. 3:**
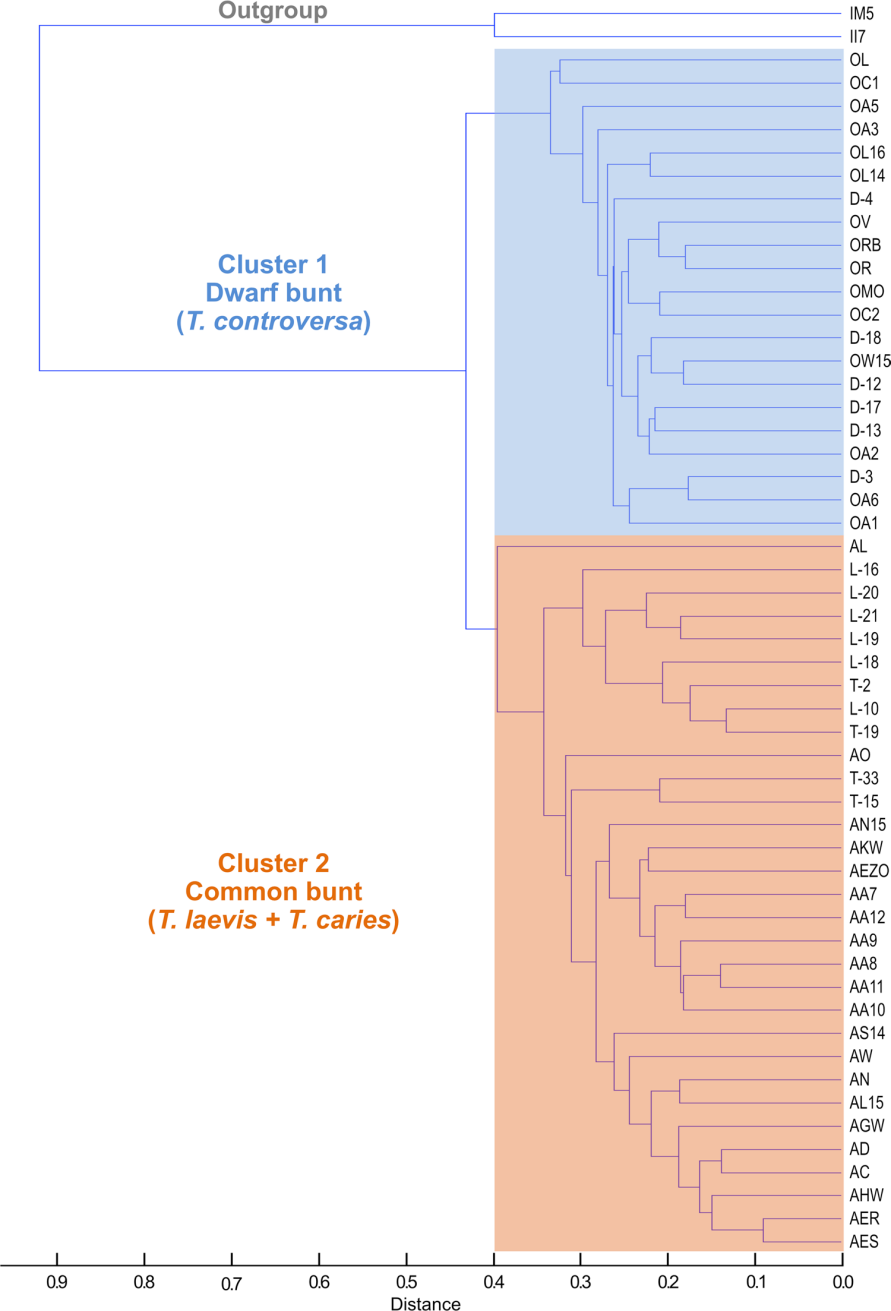
Hierarchical cluster analysis of 54 *Tilletia* specimens (reference set) displayed in a dendrogram based on the consensus spectra of nine single spectra per specimen. Two karnal bunt specimens (*T. indica*) served as outgroup. Cluster 1 included dwarf bunt specimens (*T. controversa*, marked in blue, top), cluster 2 included common bunt specimens (*T. caries* and *T. laevis*, marked in orange, bottom)

**Fig. 4:**
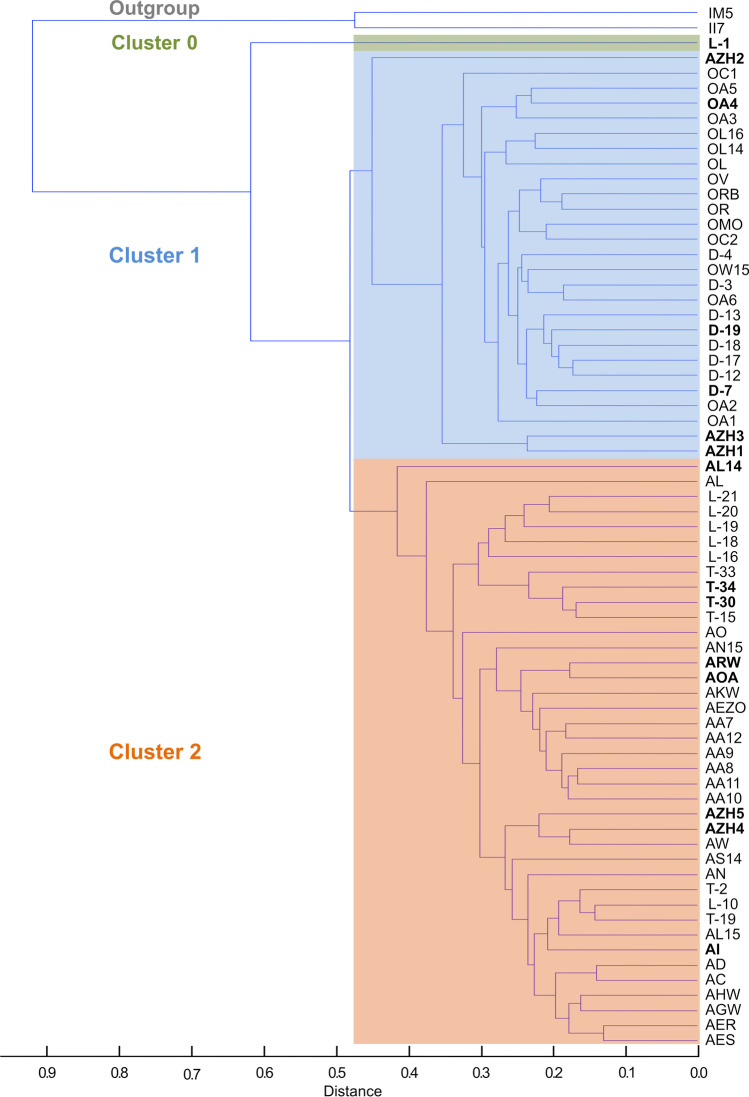
Hierarchical cluster analysis of 69 *Tilletia* specimens (including 15 test specimens) displayed in a dendrogram based on the consensus spectra of nine single spectra per specimen. Two karnal bunt specimens (*T. indica*) served as outgroup. Cluster 1 mainly included dwarf bunt specimens (*T. controversa*, marked in blue, middle), cluster 2 included common bunt specimens (*T. caries* and *T. laevis*, marked in orange, bottom). Cluster 0 included one *T. laevis* specimen (marked in green, top). The 15 test specimens are highlighted in bold type

In total, teliospores of 69 *Tilletia* specimens (52 reference specimens, 15 test specimens, and 2 *T. indica* specimens as outgroup) were analyzed by MALDI-TOF MS using our optimized protocol. The hierarchical cluster analysis (HCA) dendrogram shown in Fig. 3 visualizes the clustering of the 52 reference samples. The two karnal bunt specimens (*T. indica* IM5 and II7) serving as outgroup in the HCA were clearly separated from all common and dwarf bunt reference samples. The reference samples formed two major clusters. Cluster 1 included all specimens identified as *T. controversa*. Cluster 2 included all *T. caries* and *T. laevis* specimens. Adding the 15 test specimens (bold type) to the HCA resulted in the dendrogram depicted in Fig. 4. The three specimens belonging to the test specimen category 3 and another three belonging to category 1 clustered together with *T. controversa* specimens in cluster 1. The four remaining specimens out of category 1 plus the four belonging to category 2 grouped together with reference samples of *T. caries* and *T. laevis* in cluster 2. The one test sample L-1 out of category 4 formed a clearly separated branch of its own that was basal to cluster 1 and 2 and was assigned as cluster 0 in Fig. 4.

### Discriminant analysis of principal components used as an alternative clustering method

**Fig. 5:**
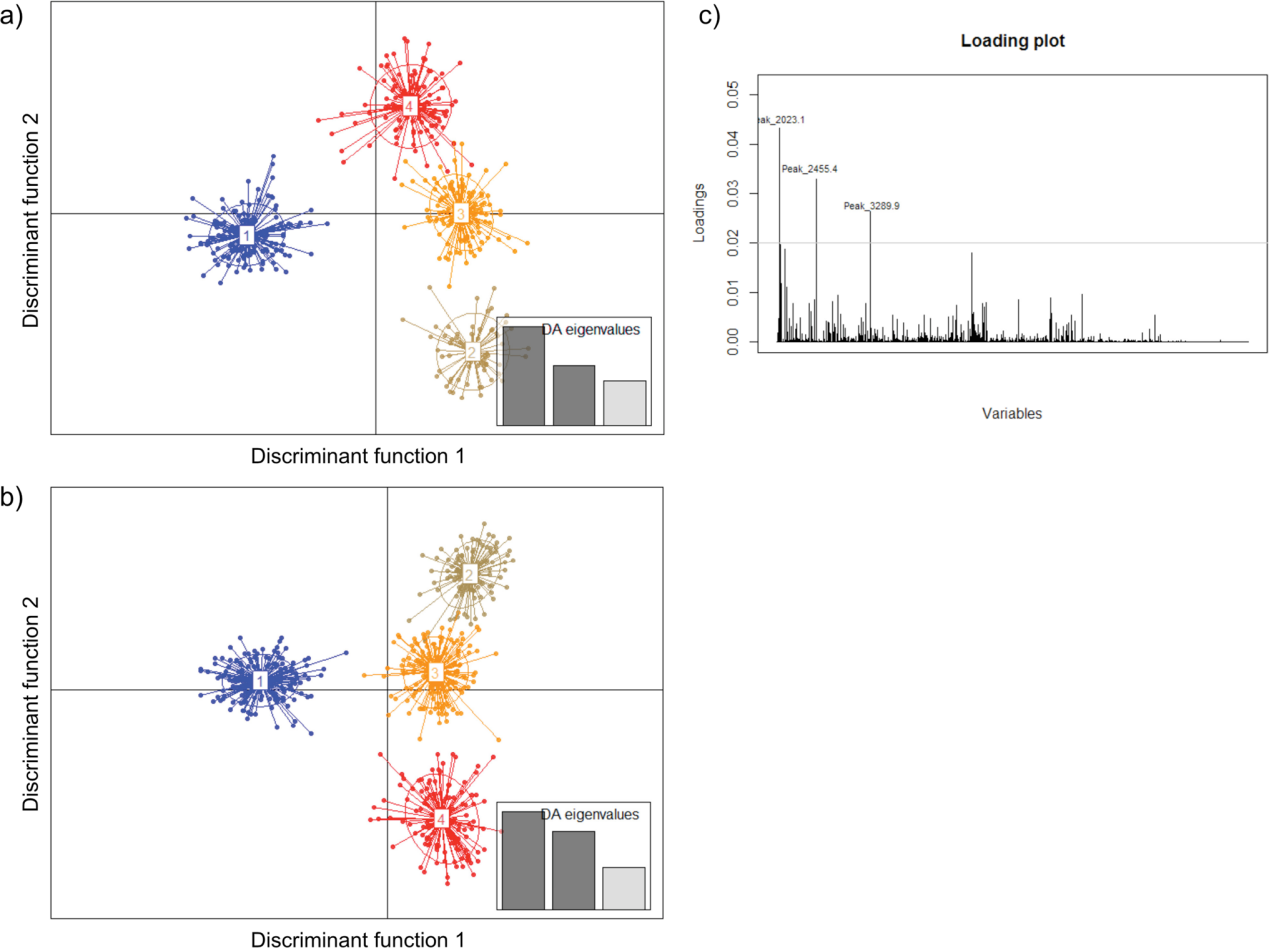
Scatterplot of a discriminant analysis of principal components (DAPC) performed with each of nine single MALDI-TOF mass spectra per specimen using teliospores from **a** 52 *Tilletia* specimens (reference set) and **b** 67 *Tilletia* specimens (reference set plus test specimens); **c** loading plot visualizing major peak differences between group 1 and groups 2–4

Separation of the *Tilletia* specimens by DAPC was chosen as an alternative approach. Grouping was based on nine single mass spectra from each specimen. Grouping of all 69 specimens from the current study resulted in a scatter plot that clearly separated the two *T. indica* specimens from the other bunt species but lacked resolution in all other specimens (see Online Resource Fig. S1). To increase the resolution, the outgroup was excluded to perform further DAPC analyses with three discriminant components. Under this condition, DAPC allocated the 52 reference specimens to four distinct groups as demonstrated in the scatter plot shown in Fig. 5a. Based on the *F*-statistics, the height difference between the first and second bar of the calculated DA eigenvalues indicated a highly significant separation of group 1 comprising almost all *T. controversa* specimens from the other three groups in the scatter plot associated with the first discriminant function (Büyüköztürk and Çokluk-Bökeoǧlu 2008). The lower second bar in DA eigenvalues confirmed some overlaps between the remaining groups 2, 3, and 4 where all *T. caries* and *T. laevis* specimens clustered (see also Table 3).

Density plots of individually performed pairwise DAPCs visualize the clear separation of the pairings of groups 1 and 2, 1 and 3 as well as 2 and 3, in contrast to all pairings including group 4 showing reduced distances of the plots (see Online Resource Fig. S2). Each pair showed a specific major peak profile (Loading plots) that was crucial for the separation of DAPC groups. The loading plot produced from the data of 52 reference specimens showed that the major peaks at *m/z* 2023.1, 2455.4, and 3289.9 were the decision criteria for the discrimination of *T. controversa* (group 1) and *T. caries/T. laevis* (groups 2–4, see Fig. 5c).

When spectra of the 15 test specimens were added to the analysis, the scatter plot of the DAPC looked similar to the previous analysis of reference specimens (compare Fig. 5a with Fig. 5b). Beside the allocation of the additional samples to the four groups (test specimens highlighted in bold type, see Table 3), few changes regarding the reference set specimens became obvious. One *T. laevis* (L-16) and one *T. caries* (T-33) specimen (group 2) as well as *T. caries* specimens AGW and AS14 (group 3) increased the number of single spectra in the respective groups now comprising more than 66%. In contrast, one or two single spectra of specimens OA1, OA2 (both group 1), L-18 (group 2), AO, and T-15 (both group 4) were transferred to other groups when test specimens were integrated with the analysis. The greatest shift was observed for specimen OA6. The nine single spectra were split between the same two groups (1 and 4) in both analyses. However, the change of allocation of two single spectra from group 1 to group 4 and 2 resulted in a new assignment of reference specimen OA6 to group 4 (see Table 3).

### Classification of the 15 test specimens and evaluation of the MALDI-TOF MS method

Finally, the 15 test specimens were classified as 12 common bunt species (*T. caries/T. laevis*) and 3 dwarf bunt species (*T. controversa*), combining the three identification criteria, morphology, germination behavior, and MALDI-TOF MS (Table 4). HCA clustering and DAPC grouping were summarized in the MALDI-TOF MS classification as dwarf bunt or common bunt because no unambiguous species allocation was possible on the basis of the mass spectrometry data equivalent to germination behavior. Comparing the final species classification with the individual results of species determination by MALDI-TOF MS, four test specimens, namely AZH1, AZH2, AZH3, and L-1, were falsely not classified as *T. caries* or *T. laevis* by HCA. On the other hand, by DAPC, only one of the test specimens (AZH3) was falsely classified as *T. controversa*. However, additionally, three *T. controversa* reference specimens, namely D-3, D-4, and OA6, clustered with seven *T. caries* specimens by DAPC, indicating a false classification as *T. caries*. In Table 5, the number of the false classified specimens by MALDI-TOF MS and the corresponding accuracy in percentage for each analysis method, HCA and DAPC, evaluated individually as well as combined, is shown. The combined final classification comprised the highest accuracy of 100.00% for the reference set and 98.51% for all specimens including the test set, respectively, as compared with both single MALDI-TOF MS data analysis methods.

**Table 4:** Classification of the 15 test specimens based on three different criteria

Test specimen	Sample no	Morphology^a^	Germination^b^	HCA cluster^c^	DAPC group^d^	MALDI-TOF MS^e^	Final classification
Category 1	AI	*T. laevis* > *T. caries*	Common bunt	2	3	Common bunt	Common bunt
	ARW	*T. caries* > *T. laevis*	Common bunt	2	3	Common bunt	Common bunt
	AZH1	*T. caries* > *T. laevis*	Common bunt	1	3	Common bunt	Common bunt
	AZH2	*T. caries* > *T. laevis*	Common bunt	1	2	Common bunt	Common bunt
	AZH3	*T. caries* > *T. laevis*	Common bunt	1	1	Dwarf bunt	Common bunt
	T-30	*T. caries* > *T. laevis*	Common bunt	2	2	Common bunt	Common bunt
	T-34	*T. caries* > *T. laevis*	Common bunt	2	2	Common bunt	Common bunt
Category 2	AL14	*T. caries* > *T. controversa*	Common bunt	2	4	Common bunt	Common bunt
	AOA	*T. caries* > *T. controversa*	Common bunt	2	3	Common bunt	Common bunt
	AZH4	*T. caries* > *T. controversa*	Common bunt	2	3	Common bunt	Common bunt
	AZH5	*T. caries* > *T. controversa*	Common bunt	2	3	Common bunt	Common bunt
Category 3	D-7	*T. caries* > *T. controversa*	Dwarf bunt	1	1	Dwarf bunt	Dwarf bunt
	D-19	*T. controversa* > *T. caries*	Dwarf bunt	1	1	Dwarf bunt	Dwarf bunt
	OA4	*T. controversa* > *T. caries*	Dwarf bunt	1	1	Dwarf bunt	Dwarf bunt
Category 4	L-1	*T. laevis*	-	0	2	Common bunt	Common bunt

^a^Morphological discrimination by 7 experts. Majority > minority

^b^Species specific germination behavior at 5 °C and 15 °C (dwarf bunt only 5 °C, common bunt at both temperatures)

^c^Clustering based on MALDI-TOF MS data analysis by hierarchical cluster analysis (HCA); 1 *T. controversa*, 2 *T. caries*/*T. laevis*, 0 outgroup

^d^Clustering based on MALDI-TOF MS data analysis by discriminant analysis of principal components (DAPC); 1 *T. controversa*, 2 *T. caries*/*T. laevis*, 3 *T. caries*, 4 *T. caries/T. controversa*

^e^Classification by MALDI-TOF MS (DAPC > HCA)

**Table 5:** Accuracy of the new developed MALDI-TOF MS method to classify *Tilletia* species to common and dwarf bunt using teliospores

		MALDI-TOF MS		
		HCA	DAPC	Final classification
Reference set (*N* = 52)	False classification^a^ (quantity)	0	2	0
	Accuracy (%)	100.00	96.15	100.00
All specimens incl. test set (*N* = 67)	False classification (quantity)	4	4	1
	Accuracy (%)	94.03	94.03	98.51

^a^Classification results were referred to the germination test, except L-1 which was referred to as the unequivocal morphological identification

## Discussion

### Reference material and test specimens

In seed testing, the causal agents of common bunt and dwarf bunt of wheat are identified based on the morphological characteristics of their teliospores such as reticulation of the spore surface, diameter and height of the muri, and presence or absence of hyaline, gelatinous sheaths (ISTA 1984; Vánky 2012). In the present study, it was attempted to identify 67 *Tilletia* specimens combining morphological characters with germination behavior of teliospores (Meiners and Waldher 1959) as a baseline for testing and establishing MALDI-TOF MS as an alternative identification method. Doing this, 52 specimens could be unequivocally determined to species level as *T. caries*, *T. laevis*, or *T. controversa*, respectively. Some conflicts remained in morphological identification of 15 species, which constituted a set of interesting test samples to examine the suitability of the developed MALDI-TOF MS method. Fourteen of them were inconsistently determined as *T. caries*, *T. laevis*, and *T. controversa* based on the morphological characteristics. Due to a broad range of variability in distinguishing characteristics, especially in spore wall markings and natural hybridization, differentiation of closely related *T. caries*, *T. laevis*, and *T. controversa* is not always that clear (Holton and Kendrick 1956; Silbernagel 1964; Hess and Trione 1986). But all these 14 specimens could be reliably classified as one of the common bunt species, *T. caries* and *T. laevis* or the dwarf bunt species *T. controversa* according to their germination behavior. The remaining test specimen, L-1, morphologically uniquely defined as *T. laevis*, did not germinate and could therefore not be assigned to the reference set (see Table 2).

### Method development

Originally, MALDI-TOF MS was developed to identify bacteria, followed by application to clinically relevant filamentous fungi (Wieser et al. 2012). The first analyzed fungal spores belonged to *Ascomycota* and *Oomycota*, where no pretreatment was necessary to extract proteins and peptides in relevant amounts and quality (Chen and Chen 2005; Chalupová et al. 2012). Teliospore cell walls of several *Basidiomycota*, in particular smut fungi, are thick-walled consisting of several spore wall layers. Therefore, a pretreatment is often necessary in order to liberate proteins and peptides for subsequent MALDI-TOF MS analyses (Taskova et al. 2006; Piepenbring 2009). In the current study, subproteomic mass spectra of *T. caries* and *T. controversa* mycelia were successfully generated using the sample preparation protocol of Cassagne et al. (2011), which has frequently been used for MS profiling of filamentous fungi and yeasts (Lau et al. 2013; Usbeck et al. 2013; Lauterbach et al. 2017; Wigmann et al. 2019). However, these protocols did not deliver high-quality mass spectra when using teliospores of *Tilletia* species without any pretreatment. Previous studies demonstrated that mechanical disruption of fungal spores resulted in higher quantities of proteins as compared to the concentrations obtained by spontaneous protein leakage (Banowetz and Doss 1994). In the meantime, several studies optimized the procedure using bead-beating homogenizers, e.g., for conidia of *Aspergillus* and *Penicillium* (Hettick et al. 2008a, b) and for clinical mold isolates including different basidiomycetes (Luethy and Zelazny 2018). Sulc et al. (2009) and Beinhauer et al. (2016) demonstrated that in addition to the quality of the mechanical disruption of spores, also the number of fungal spores per treatment must be at an optimum to yield good mass spectrometry results. In the current study, concentrations between 1 and 5 mg of teliospores were tested, resulting in an optimal signal-to-noise ratio at 3 mg, which is equivalent to ca. 10^6^ teliospores of *Tilletia* spp. (data not shown). Beside the concentrations of proteins and byproducts, also the way in which samples are applied to the MALDI-TOF MS target plate has a considerable influence on the quality of spectra. Comparing different preparation techniques, the sandwich method, applying matrix-aliquot-matrix, yielded the most satisfactory mass spectra in combination with α-CHCA matrix, which is tolerant towards volatile contaminants (Kussmann et al. 1997), which are supposed to exist in *Tilletia* teliospores (Holton et al. 1968). Using the developed method for MALDI-TOF MS-based teliospore analysis, we were able to create reproducible mass spectra of one specimen each of *T. caries* and *T. controversa* which could clearly separate both species. Comparison of spectra produced from their mycelia showed that the teliospore-based spectra produced similar numbers of peaks, albeit with lower intensities, but still strong enough to be clearly resolved by the MASCAP algorithm (Mantini et al. 2010). Also, the results of DAPC showed a clear separation of the two species along with a discriminant function (Fig. 2a) based on the major peak differences visualized in the loading plots (Fig. 2b). This means that teliospore-based analysis equals its mycelium-based equivalent in quality with the advantage of skipping the time-consuming germination and cultivation step.

### Classification of reference specimens using HCA and DAPC

Analysis of the 52 *Tilletia* reference specimens alone aimed at obtaining a general grouping that allowed the distinction of the three species. The results presented here clearly demonstrate that the interpretation of mass spectra and the resulting species identification strongly depends on the method used for data clustering. HCA and DAPC were used for the clustering of spectra in the current study. Several previous studies have used either one or both analysis methods to differentiate closely related taxa of bacteria, fungi, or yeasts (Hettick et al. 2008a, b; Usbeck et al. 2013; Kehrmann et al. 2016; Lauterbach et al. 2017; Wigmann et al. 2019). Similar to the findings of the current study, most authors found differences in the clustering results produced by the two methods. The reason for the occurrence of such differences is the use of data that differ in their degree of variability. HCA creates clusters by calculating distances based on consensus spectra that are an average of several single spectra and do not reflect the variance within samples. In contrast, DAPC separates samples on the basis of the maximum variance found between the single spectra taken from each sample (Kehrmann et al. 2016). DAPC therefore can be used to identify single outliers probably produced by technical variation during sample preparation or during the measurement, which may have an influence on the clustering of the mean spectra used in HCA.

All in all, the clustering of mean spectra using HCA resulted in a clear separation of the reference specimens into a group representing *T. controversa* and a second group representing *T. caries* together with *T. laevis* congruent to the classification by germination behavior. Using DAPC, the 52 reference specimens were separated into four groups, which consisted of *T. controversa* only, *T. caries* only, *T. caries* together with *T. laevis*, and *T. caries* together with *T. controversa*. The highest variation of common mass spectra profiles between the groups was identified when choosing four groups using Bayesian information criterion (BIC) included in DAPC using adegenet 2.0.0 (Jombart and Collins 2015). Three or two groups would have been obvious when expecting three different species or two groups representing the causal agents of common bunt and dwarf bunt, respectively, but it seems like there was more variation in the MALDI-TOF MS profiles between the analyzed samples. At first sight, DAPC seemed to be able to distinguish the *T. caries* from the *T. laevis* specimens in contrast to HCA, but a closer look reveals that no further discrimination between the two species was possible with the applied protocol. The main difference between the DAPC groups comprising *T. caries* and *T. laevis* was the origin of the specimens, supporting the hypothesis of regional but not species-derived clustering. The specimens *T. laevis* “L” and *T. caries* “T” originated from the USA and clustered together in contrast to the European *T. caries* “A” samples. In contrast, no clustering according to geographic origin – USA vs. Europe – could be identified in the specimens used for *T. controversa*. More samples, especially *T. laevis* specimens from another origin, are needed to further examine the occurrence of species-specific or region-dependent clustering of *Tilletia* species. All in all, the vast majority of *T. controversa* specimens, except two out of 21, were separated from *T. caries* and *T. laevis* by DAPC. In total, the clustering of the 52 reference specimens by HCA was more specific. However, both analysis methods were not able to distinguish the common bunt species *T. caries* and *T. laevis* based on mass spectra created by MALDI-TOF MS supporting the clustering obtained by germination behavior and initial results obtained by genome sequencing, which indicated a high degree of genomic identity of *T. caries* and *T. laevis* (Sedaghatjoo et al. 2021b, submitted). Several studies already query the common taxonomic classification of the three *Tilletia* species (Russell and Mills 1994; Carris et al. 2007), especially the separation of *T. caries* and *T. laevis* as two distinct species (Shi et al. 1996). The groupings of specimens obtained by DAPC could also indicate a conspecific status of *T. caries* and *T. laevis*. Both agents of the common bunt disease share the same physiological features of teliospores and do not differ in the host range and distribution or their pathology towards wheat (Leppik 1970; Wilcoxson and Saari 1996). Several studies also showed that *Tilletia* species can hybridize. As a consequence, genetic exchange between species could lead to highly variable morphological characteristics (Holton and Kendrick 1956; Silbernagel 1964) which would also be represented in the variability of the proteome. Moreover, the occurrence of different morphotypes is quite common among microorganisms (Armaleo and Clerc 1991; Gilbert et al. 2018). It has been demonstrated that the presence or absence of a single gene or even few SNPs can suffice to create two or more morphotypes of one species (Gilbert et al. 2018). In addition, also the microenvironment and exogenous factors can influence the morphogenetic characteristics of fungi (Lin et al. 2015). It is currently unknown which genes are responsible for the expression of the morphological characteristics such as shape and height of ornamentation of the cell wall of *Tilletia* teliospores (Nguyen et al. 2019; Sedaghatjoo et al. 2021b). Since it appears that micromorphological spore characteristics are the only possibility to distinguish the two common bunt species *T. caries* and *T. laevis*, we suggest that those two taxa could be treated as two morphotypes of the same species.

#### Classification of test specimens and accuracy of the MALDI-TOF MS method

Focusing on the 15 *Tilletia* spp. test specimens in this study, the main differences between the two analytical methods became obvious with specimens AZH1, AZH2, and L-1. These clustered together *T. caries* and *T. laevis* by DAPC but together with the *T. controversa* cluster or even as outgroup when analyzed by HCA (see Table 4). Referring to the germination behavior and the morphological identification of specimens AZH1 and AZH2, the results obtained by DAPC appear more reliable. The loss of viability of the L-1 teliospores, depicted by lack of germination, may have caused an effect on the proteome spectra, which became obvious by HCA. Enzymes potentially essential for teliospore germination could be reduced during a lifetime (Schauz 1968). Nonviable *Tilletia* teliospores compared with viable ones did not show lipase or glucosidase activity and lack acid phosphatase enzymes (Yu et al. 1984; Chastain and King 1990). Talbot et al. (2010) showed that in some cases, MALDI-TOF MS peak information of nonviable samples differ from the representative peaks in spectra of active spores. Such peak variations could be crucial considering the processing of the mass spectra for discrimination of the species by HCA. Creating a consensus spectrum of a nonviable sample may reduce weak signals still detectable by DAPC focusing on the variances. The grouping of specimen L-1 along with the other *T. laevis* species by DAPC (see Table 3) indicates that enough species-specific mass spectra of nonviable samples can be generated by MALDI-TOF MS. Other challenging test samples were specimens AI and D-7. Both were difficult to identify morphologically as reflected by the conflicting determinations of these specimens by the experts. Based on our developed MALDI-TOF MS method, the specimen AI was determined as one of the common bunt species, but identification to species level was impossible. On the other hand, the new MALDI-TOF MS method grouped specimen D-7 as *T. controversa* in accordance with its germination behavior confirming the potential of MALDI-TOF MS to distinguish *Tilletia* specimens comprising varying morphological characteristics.

Finally, summing up the potential false classifications by MALDI-TOF MS analysis methods considering the whole set of 67 *Tilletia* specimens, both HCA and DAPC clustered four specimens incorrectly (AZH1, AZH2, AZH3, L-1 vs. AZH3, D-3, D-4, OA6), while only one specimen (AZH3) remained heterogeneous (see Table 1). Both MALDI-TOF MS clustering analyses classified this specimen as *T. controversa* in contrast to the specific germination behavior and morphological species identification classified it as *T. caries* or *T. laevis*. Therefore, the newly developed MALDI-TOF MS method resulted in an accuracy of 98.51% (Table 5) and hence behaves similar to the germination behavior-based determination, but is much faster. By integrating HCA and DAPC, taking the similarities as well as the variances of the MALDI-TOF MS spectra into account, the best results in discrimination of the studied *Tilletia* species can be obtained.

In conclusion, in this study, we developed and optimized the MALDI-TOF MS method for the differentiation of three important wheat pathogens applying teliospores isolated from bunt balls, which has proven to be a useful and fast tool. However, *T. caries* and *T.* laevis, the causal agents of common bunt, cannot be individually discriminated, but separated from *T. controversa,* the causal agent of dwarf bunt. This supports a potentially conspecific status of *T. caries* and *T. laevis* or even two morphotypes of one common species causing identical disease symptoms and sharing the same germination requirements along with a related protein composition, shown in this study. To easily screen new *Tilletia* specimens or a large number of specimens by MALDI-TOF MS in the future, validated main spectrum profiles (MSP) of each *Tilletia* species are needed for a fast and specific species identification. These specific MSPs could be produced and deposited in a database like the MSI platform (Normand et al. 2017) for further studies. Our developed MALDI-TOF MS method can be helpful in testing *Tilletia* bunt balls collected during field inspections, especially with regard to quarantine regulations or for breeding applications.

## Supplementary Information

Below is the link to the electronic supplementary material.

Supplementary file1 (PDF 270 KB)

## Data Availability

All specimens analyzed during the current study are stored and are available at the official seed testing laboratory of the Bavarian State Research Center for Agriculture (Freising, Germany) as well as at the Julius Kühn-Institute (JKI) – Federal Research Centre for Cultivated Plants (Braunschweig, Germany). Samples numbered 31–38, 41–47, and 64–69 were supplied by USDA (Agricultural Research Service—U.S. Department of Agriculture) and are available upon request. The mass spectrometry proteomics data are available at the ProteomeXchange Consortium home page under http://proteomecentral.proteomexchange.org/cgi/GetDataset with the dataset identifier PXD030401.
