# Supplementary material for: Discrimination of Tilletia controversa from the T. caries/T. laevis complex by MALDI-TOF MS analysis of teliospores
Source: Appl Microbiol Biotechnol. 2022 Jan 17;106(3):1257–78. doi: 10.1007/s00253-021-11757-2 (PMC8816839; doi:10.1007/s00253-021-11757-2)

Discrimination of *Tilletia controversa* from the *T. caries* / *T. laevis* complex by MALDI-TOF MS analysis of teliospores

Applied Microbiology and Biotechnology

Monika K. Forster, Somayyeh Sedaghatjoo, Wolfgang Maier, Berta Killermann, Ludwig Niessen*

*Corresponding author

Affiliation: Chair of Microbiology, TUM School of Life Sciences, Technical University ofMunich, Gregor-Mendel-Str. 4, 85354 Freising, Germany

Email: ludwig.niessen@tum.de

Supplementary Information

Fig. S1 Scatterplot of a discriminant analysis of principal components (DAPC) performed with each nine single MALDI-TOF mass spectra per specimen using teliospores from 69 *Tilletia* specimens. Group 5 includes two specimens of *T. indica* (outgroup). Group 3 mainly includes *T. controversa* specimens while common bunt specimens are distributed among group 1, 2, and 4

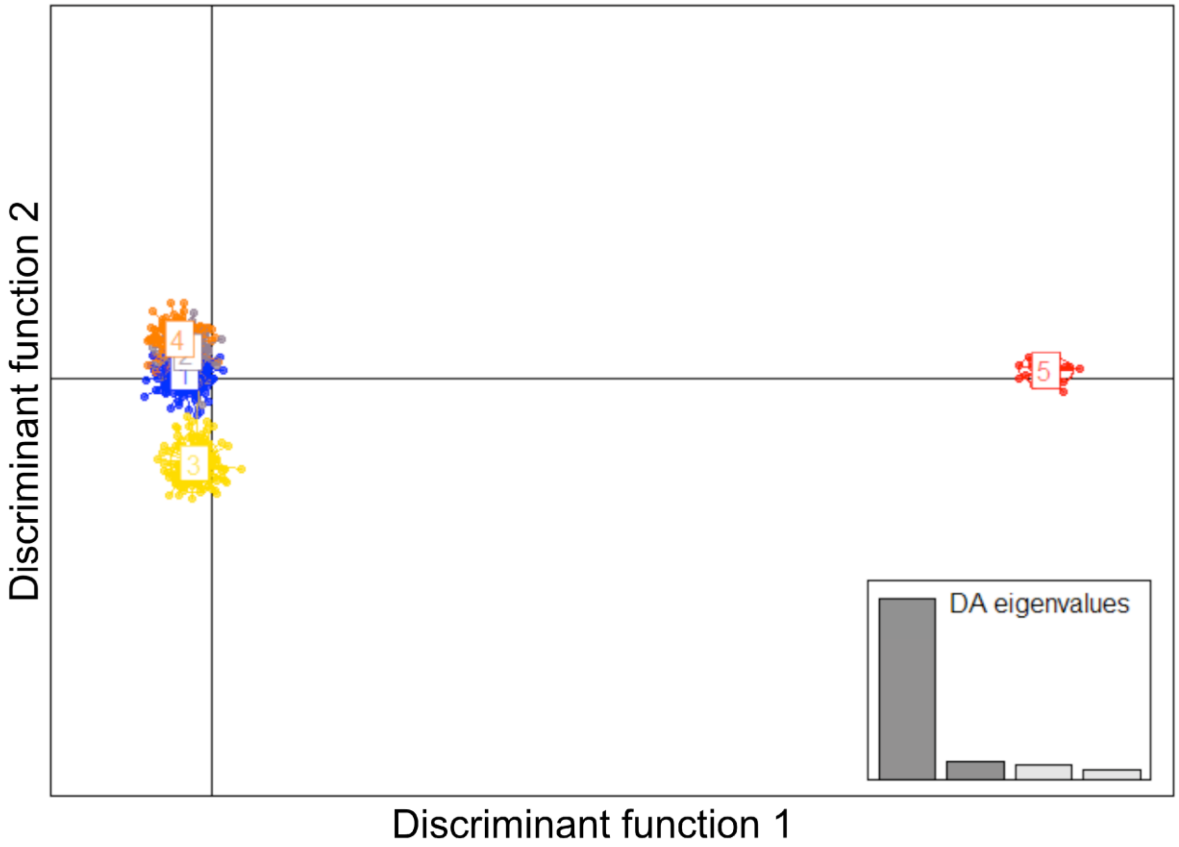

Fig. S2 Pairwise discriminant analysis of principal components (DAPC) performed with single MALDI-TOF mass spectra of the 52 *Tilletia* species included in the corresponding DAPC group (reference specimens, Figure 4a). Visualization of the major peaks crucial for the separation of the *Tilletia* species in four groups and the individual density plot for each group pair. a) Separation of groups 1 and 2; b) separation of groups 1 and 4; c) separation of groups 1 and 3; d) separation of groups 2 and 4; e) separation of groups 2 and 3; f) separation of groups 3 and 4

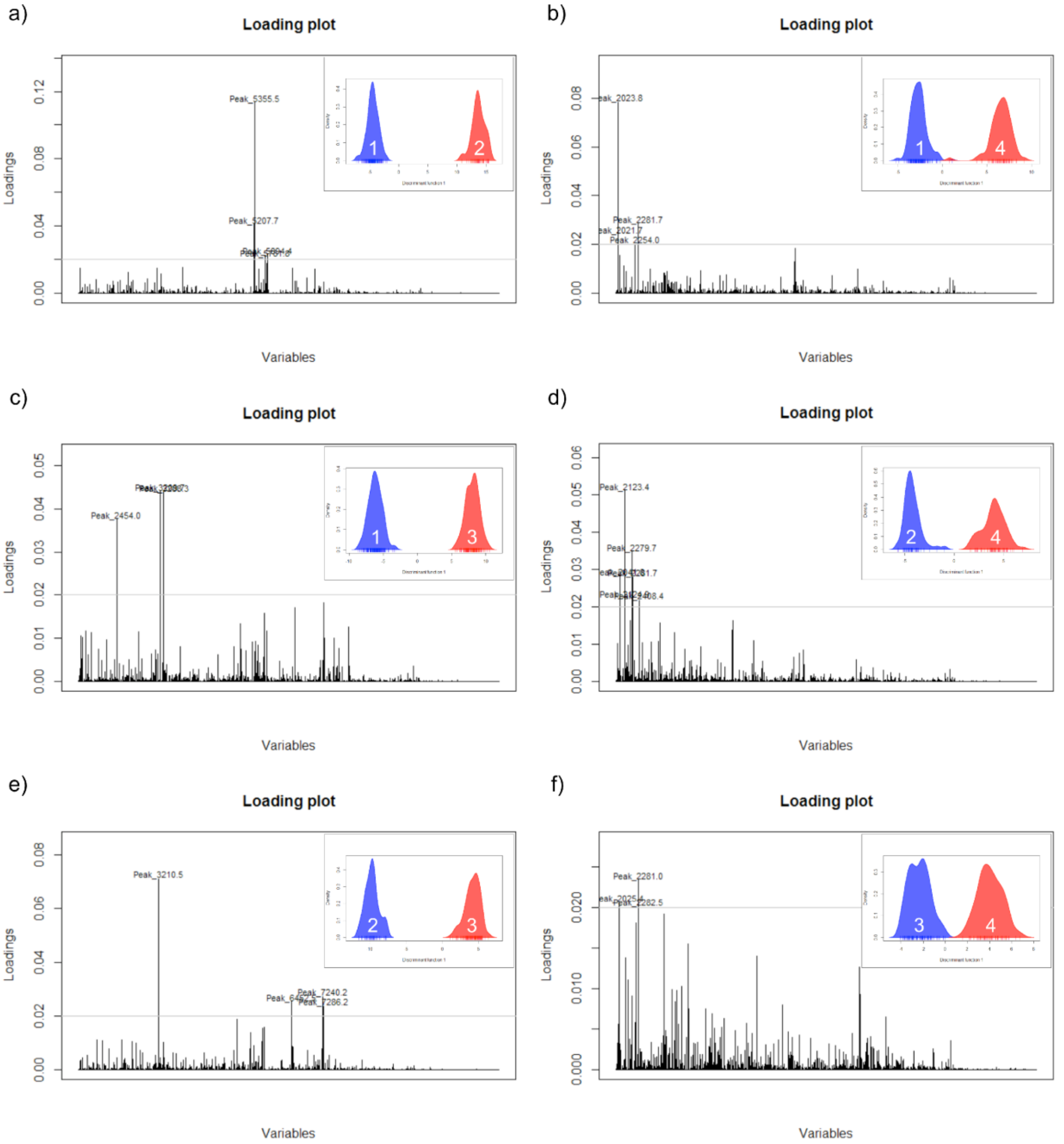

Supplement: Supplementary file 1 — Supplementary file1 (PDF 270 KB) [file 253_2021_11757_MOESM1_ESM.pdf]
